# Whole-genome shotgun sequencing unravels the influence of environmental microbial co-infections on the treatment efficacy for severe pediatric infectious diseases

**DOI:** 10.3389/fmicb.2024.1308871

**Published:** 2024-01-24

**Authors:** Chengxin Li, Jing Song, Qihong Chen, Dandan Ge, Qiyuan Li, Yungang Yang

**Affiliations:** ^1^Department of Pediatrics, Pediatric Key Laboratory of Xiamen, The First Affiliated Hospital of Xiamen University, Institute of Pediatrics, School of Medicine, Xiamen University, Xiamen, China; ^2^National Institute for Data Science in Health and Medicine, School of Medicine, Xiamen University, Xiamen, China

**Keywords:** shotgun sequencing, severe infection, microbiome, pediatric, co-infection

## Abstract

**Background:**

The microbiome plays a pivotal role in mediating immune deviation during the development of early-life viral infections. Recurrent infections in children are considered a risk factor for disease development. This study delves into the metagenomics of the microbiome in children suffering from severe infections, seeking to identify potential sources of these infections.

**Aims:**

The aim of this study was to identify the specific microorganisms and factors that significantly influence the treatment duration in patients suffering from severe infections. We sought to understand how these microbial communities and other variables may affect the treatment duration and the use of antibiotics of these patients with severe infections.

**Method:**

Whole-genome shotgun sequencing was conducted on samples collected from children aged 0–14 years with severe infections, admitted to the Pediatrics Department of Xiamen First Hospital. The Kraken2 algorithm was used for taxonomic identification from sequence reads, and linear mixed models were employed to identify significant microorganisms influencing treatment duration. *Colwellia*, *Cryptococcus*, and *Citrobacter* were found to significantly correlate with the duration of clinical treatment. Further analysis using propensity score matching (PSM) and rank-sum test identified clinical indicators significantly associated with the presence of these microorganisms.

**Results:**

Using a linear mixed model after removed the outliers, we identified that the abundance of *Colwellia*, *Cryptococcus*, and *Citrobacter* significantly influences the treatment duration. The presence of these microorganisms is associated with a longer treatment duration for patients. Furthermore, these microorganisms were found to impact various clinical measures. Notably, an increase in hospitalization durations and medication costs was observed in patients with these microorganisms. In patients with *Colwellia*, *Cryptococcus*, and *Citrobacter*, we discover significant differences in platelets levels. We also find that in patients with *Cryptococcus*, white blood cells, hemoglobin, and neutrophils levels are lower.

**Conclusion:**

These findings suggest that *Colwellia, Cryptococcus*, and *Citrobacter*, particularly *Cryptococcus*, could potentially contribute to the severity of infections observed in this cohort, possibly as co-infections. These microorganisms warrant further investigation into their pathogenic roles and mechanisms of action, as their presence in combination with disease-causing organisms may have a synergistic effect on disease severity. Understanding the interplay between these microorganisms and pathogenic agents could provide valuable insights into the complex nature of severe pediatric infections and guide the development of targeted therapeutic strategies.

## 1 Introduction

Children are known to be more susceptible to severe infections, and these infections can have significant physical and economic consequences for patients (Tiru et al., 2015; Sultana et al., 2021). Therefore, accurate identification of pathogenic bacteria is crucial for effective treatment (Brook, 2017). Metagenomic next-generation sequencing (NGS) has emerged as a valuable clinical tool for diagnosing severe infections, particularly those caused by rare pathogens (Wu et al., 2019; Consensus Group of Experts on Application of Metagenomic Next Generation Sequencing in The Pathogen Diagnosis in Clinical Moderate and Severe Infections et al., 2020; Wang et al., 2022). In terms of technological capabilities, shotgun sequencing offers a more powerful tool compared to 16S rRNA sequencing (Bender and Dien, 2018). Its higher resolution and functional profiling capability make it an advantageous approach, particularly as very few studies have applied metagenomic sequencing in pediatric populations.

Shotgun sequencing can simultaneously detect multiple pathogens such as bacteria, fungi, and viruses, thereby broadening the detection spectrum and enhancing the overall pathogen detection rate (Chiu and Miller, 2019). In this study, we aimed to utilize whole-genome shotgun sequencing and taxonomic identification algorithms to identify the microbial species present in samples collected from pediatric patients with severe infections. We hypothesize that the microbiomes of these patients may play a crucial role in influencing disease progression and treatment outcomes.

Therefore, our goals were twofold: (1) we aimed to characterize and analyze the microbiomes present in the infection samples of pediatric patients with severe infections, using shotgun sequencing. This approach allows us to identify and understand the roles and interactions of these microorganisms within the host, which could potentially be contributing to the severity of the infections, and (2) to explore the relationship between microbiomes and clinical characteristic, with a particular focus on identifying microorganisms that may contribute to disease severity through their association with various clinical indicators. This comprehensive analysis of the microbiome composition in different sample types will provide valuable insights into the potential origins of severe infections in children and may contribute to the development of targeted prevention and treatment strategies.

## 2 Materials and methods

### 2.1 Patients

123 hospitalized children aged 0–14 years who were admitted to the pediatric department of Xiamen First Hospital with severe infections who were tested for metagenomics next-generation sequencing from 2020 to 2022 were recruited. Children with severe infections exhibit the following characteristics: (1) persistent inflammation (continual presence of fever or abnormal laboratory findings such as procalcitonin (PCT), C-reactive protein (CRP) or sustained white blood cell counts) or disruption of normal activities for more than 1 week (Alkhater, 2009), (2) meeting diagnostic criteria for severe pneumonia (National Health Commission of the People’s Republic of China, State Administration of Traditional Chinese Medicine, 2019), (3) concurrent organ failure (Dellinger et al., 2013), and (4) those with extracorporeal metagenomic specimens. The Medical Ethics Committee of Xiamen University approved the study, including all procedures (recruitment of participants and all experimental protocols). Written informed consent was obtained from patients selected from Xiamen First Hospital. A total of 123 patients with severe infections were diagnosed by pathological examination. All participants were Chinese residents of Fujian Province, China.

### 2.2 Data collection for clinical information

The patient’s private information, such as name and medical record number, has been removed from the system. The unique identifier for each patient is a 6-character code composed of letters and numbers. The data under investigation does not contain any personally identifiable information, and all sensitive fields have been anonymized. Clinical information includes visit time, age, sex, treatment time, the cost of medicine, type of antibiotics, diagnosis and laboratory indexes. Laboratory indexes consist in white blood cell count (WBC), neutrophil percentage (NE%), hemoglobin levels (HGB), platelet count (PLT), procalcitonin (PCT), albumin, transaminase, LDH and coagulation function.

### 2.3 Sample collection

For each patient, multiple samples of the same type were collected at different stages of the disease to monitor the progression and response to treatment. In addition, samples were also collected from different sites of the same patient, which might indicate possible infection sites. For instance, in cases of suspected pulmonary infection, bronchoalveolar lavage fluid was collected multiple times throughout the course of the disease. In cases where other systemic infections were suspected, a selection of samples from cerebrospinal fluid, urine, intraperitoneal effusion, and blood were collected, specifically from the sites where infection was suspected. All samples were collected according to the standard procedures described above.

(a) As for the bronchoalveolar lavage fluid (BALF), bronchoalveolar lavage was performed on every patient diagnosed with pneumonia. In localized disease, the lavage is most likely perfumed on the segment where the disease is most prominent. In diffuse disease, the right middle lobe or lingula is usually lavage. We want to instill sterilized saline which is prewarming to 37°C by 1 mL/kg aliquots. After performing sufficient suction and recovery, a 5 mL sample was collected for examination. (b) For cerebrospinal fluid, standard lumbar puncture was performed on the patient, and more than 2 mL of cerebrospinal fluid was collected. (c) Regarding urine samples, local digestion of the meatus and adult mucosa were performed with a non-foaming antiseptic solution, such as Dakin’s solution. The initial stream of the voided specifics was discarded. At least 5 mL midstream sample was collected and sent to the companies for analysis. (d) Abdominal puncture was performed to collect intraperitoneal effusion, and a minimum volume of 5 mL was obtained for analysis. (e) As for blood samples, the typical procedure involves disinfecting the venous puncture site with 2% chlorhexidine alcohol or alcoholic iodine. Subsequently, a butterfly needle is used to extract 3–5 mL of blood (the specific volume depends on the age of the individual) for testing purposes. All specimens were collected and stored in a −20°C refrigerator. Subsequently, they were collected by the corresponding company and transported under dry ice conditions to ensure proper storage and preservation during transportation.

### 2.4 Shotgun metagenomic sequencing for each sample

Total genomic DNA and RNA were extracted from five different types of samples, and both DNA and RNA samples were used for shotgun library construction. Subsequently, high-throughput sequencing was performed on the samples using four platforms, including Thermo Fisher Scientific (DA8600), Illumina NextSeq 550, MGI 200/2000, and Q-mNGS^™^3.0. The sequencing was conducted in paired-end mode with a read length of 150 base pairs (PE150). It is noteworthy that the three batches of samples were obtained from distinct companies. Whole-genome shotgun sequencing was employed to generate paired-end outputs with a read length of 150 base pairs.

### 2.5 Sequence data pre-processing

The quality control checks on all the raw sequence data by using the FastQC (Andrews, 2010) version 0.11.9 and MultiQC (Ewels, 2016) version 1.12. The raw reads were pre-processed using KneadData (McIver et al., 2018) version 0.10.0 for quality control and host sequence decontamination (based on Trimmomatic version 0.39) (Bolger et al., 2014) and Bowtie2 version 2.4.5 (Langmead and Salzberg, 2012) with the recommended human reference database. In summary, the quality control and criteria of the pre-process procedure: (1) filtering out of the human genome contaminated reads by aligning raw reads to the human reference genome (GRCh37/hg19) (2) removal of adaptor sequences using Trimmomatic (trimming: SLIDINGWINDOW:4:20 MINLEN:25) and (3) ensuring quality scores adhere to phred33 standards. Additionally, (4) Tandem Repeats Finder is not employed to locate and remove reads resembling tandem repeats.

### 2.6 Taxonomic analysis

Taxonomy assignment were performed using Kraken2 (Wood et al., 2019) version 2.7.7 with default settings. Based on the standard reference database of clade specific marker genes embracing viruses, archaea, bacteria, eukaryotes, human and carriers. In all cases we use the defaults for k-mer length, minimizer length, and minimizer spacing. Results were visualized by MEGAN6 (Beier et al., 2017) and R package phyloseq (McMurdie and Holmes, 2013). In order to reduce the differences in concentration between different samples, the raw data were converted to percentages, and OTUs with a Median Absolute Deviation (MAD) value greater than 0.5 were selected to retain those that had a significant impact on sample differences. The selected OTUs will be used for subsequent multivariate analysis to explore the differences in microbial communities among different samples.

### 2.7 Statistical analysis

#### 2.7.1 Principal component analysis (PCA)

Due to the diversity of measures and samples included in the study, we expected to observe heterogeneity in effect size estimates. To address potential batch effects between samples, we conducted a principal component analysis and identified outliers based on the first principal component (Leek et al., 2010). Based on the scores of the first principal component, we selected and removed outlier samples in downstream analyses to minimize the impact of batch effects.

#### 2.7.2 Linear mixed models

To account for the within-individual correlations of multiple samples, we used linear mixed-effects models in our statistical analysis. In our statistical analysis, we treated these multiple samples as repeated measures from the same individual and used appropriate statistical methods (mixed-effects models) to account for the within-individual correlations. Linear mixed models (De Boeck et al., 2011) were constructed via R package lme4. In order to assess the significance of differences in clinical variables across samples, the analysis of variance (ANOVA) (McHugh, 2011) with repeated measurements was used. The likelihood ratio tests with Benjamini-Hochberg FDR correction (Benjamini and Hochberg, 1995) were used to screen out non-significant models and non-significant clinical variables. Effect size was estimated using marginal and conditional (pseudo-R2) linear association between standardized variables. Linear mixed models (with random intercepts and slopes) were applied. Firstly, likelihood ratio tests (with Benjamini Hochberg correction) were applied to the simple models (no condition effects) to assess the significance of the regression coefficient and effect size was estimated by marginal and conditional (pseudo-R2). Then, likelihood ratio tests (with Benjamini-Hochberg correction) were applied to compare simple and extended models to determine whether regression coefficient differ significantly between samples. We tested a series of nested models that included the relevant clinical variables such as age group, gender, and sample type as fixed effects. In these models, the batch was incorporated as a random effect. Based on the proposed empirical importance and practical considerations, individual clinical characteristics were introduced into the model, and the final best-fit model was identified as the one with the lowest Akaike information criterion (AIC) (Liang et al., 2008) value and with statistical significance. After conducting the final screening, we developed a model that included fixed effects such as age group, gender, treatment outcome, and ID specialist antibiotics. The data source was considered as a random effect in the model, taking into account the three different batches from which the data was sourced. In analyzing the taxonomic profiles of the microbial community, we utilized a significant model to compare differences between genera. We performed separate inclusion of each genus in the model as a fixed effect to assess which genus caused the effect on the clinical variable under study. We then selected the microorganisms that were found to have a significant impact on the treatment time, which was the outcome of interest in our study. This study considered potential microorganisms that displayed consistent statistical significance from all analyses above.

#### 2.7.3 Propensity score matching (PSM)

Propensity score matching (PSM) (Hill and Reiter, 2006; Caliendo and Kopeinig, 2008) was performed to match individuals between the presence of pathogens group and the absence of pathogens group based on age group, gender, and sample types in a 1:2 ratio. By using age group, gender, and sample types as covariates for matching, we effectively control for potential confounding factors that could influence the presence of pathogens and treatment outcomes in the dataset. The 1:2 ratio in matching ensures that for each individual in the presence of pathogens group, two individuals with similar age group, gender, and sample type characteristics are selected from the absence of pathogens group, thereby creating a more comparable and representative dataset.

This careful matching process strengthens the validity of our analysis and allows for a more robust assessment of the causal impact of, *Cryptococcus*, and *Citrobacter* on treatment time and various clinical indicators in pediatric infections. By controlling for these important covariates, we can more confidently attribute any observed differences in outcomes to the presence or absence of these three pathogens.

#### 2.7.4 Wilcoxon rank-sum tests

Wilcoxon rank-sum tests were performed to assess the significance of various clinical indicators in relation to the presence or absence of the pathogens. These tests allowed for a robust comparison of clinical metrics between the groups with and without *Cryptococcus*, *Citrobacter*, and *Colwellia*.

The nonparametric rank sum tests were chosen because they do not assume a specific distribution for the data, making them suitable for analyzing clinical indicators that may not follow a normal distribution. By using rank sum tests, we could determine whether there were significant differences in clinical indicators, such as treatment duration, lymphocyte percentage (LY%), neutrophil percentage (NE%), hemoglobin levels (HGB), platelet count (PLT), and white blood cell count (WBC), between the two groups.

By combining the results of propensity score matching (PSM) with Wilcoxon rank-sum test, we were able to gain deeper insights into the potential impact of *Cryptococcus*, *Citrobacter* and *Colwellia* on various clinical outcomes in severe pediatric infections. These statistical analyses strengthen the validity of our findings and support the conclusions drawn from the study.

In the aforementioned statistical analyses, a significance threshold of *p* < 0.05 was utilized to ensure statistical significance across all conducted tests. All statistical analyses were performed using the R environment (version 4.3.0). Bash and R script implementations of the analysis are available on GitHub (https://github.com/iChronostasis/EnvMicrobePediatricTreatmentAnalysis) for academic use.

## 3 Results

### 3.1 Patient characteristics and sample analysis in severe infections

123 diagnosed severe infected patients were enrolled in our study, including 69 males and 54 females, and their average age was 3.814 ± 4.15 years. 172 samples were collected for inclusion in this analysis from three batches (Table 1). The 172 samples included in this analysis were collected from 123 patients, with 34 patients contributing multiple samples (Supplementary Table S1). The clinical information of these samples, including gender and age, was recorded at the time of sample collection. To account for the potential within-individual correlations, we compared the gender and age group using mixed-effects models that included the individual as a random effect. We did not observe any significant differences in gender or age between the three batches. (a) These three batches’ samples number were 106, 33, and 33, respectively (Supplementary Table S2). (b) Blood, bronchoalveolar lavage fluid (BALF), cerebrospinal fluid (CSF), urine, and ascites were collected from patients separately, with sample sizes of 74, 53, 36, 4, and 5, respectively (Supplementary Figure S1A). (c) The distribution of treatment time in all samples is shown in Supplementary Figure S1B. (d) Metagenomic results showed a total of 104 samples were positive, 67 were negative and 1 was not analyzed (Supplementary Figure S1C). (e) The culture results of the samples showed that a total of 50 samples were positive, 120 were negative and 2 were not analyzed (Supplementary Figure S1D).

**Table 1:** Demographic of enrolled patients.

Demographic of enrolled patients (*n* = 123)			
Gender	Male	Female	
	69	54	
Age group	4 years old and above	Under 4 years old	
	51	72	
Age	Mean	SD	
	3.814	4.15	
Marrow depression	Yes	No	NA
	23	99	1
Antibiotic treatment	Yes	No	
	121	2	
ID specialist antibiotics	Yes	No	
	78	45	

This table outlines the demographic and clinical characteristics of the 123 patients with severe infections who participated in our study. The data is categorized by sex (69 males and 54 females) and age group (51 patients aged 4 years and above, and 72 patients under 4 years old). The mean age of the patients is 3.814 years with a standard deviation of 4.15 years. In terms of clinical characteristics, 23 patients presented with marrow depression while 99 did not. One patient’s marrow depression status was not available. The source of infection was classified as pulmonary (57 patients), intracranial (19 patients), urinary tract (6 patients), and bloodstream (41 patients). The majority of patients (121 out of 123) received antibiotic treatment. The use of ID specialist antibiotics was reported in 78 patients, while 45 patients did not receive these medications.

### 3.2 Comprehensive analysis reveals high microbial diversity in pediatric infection samples

At the kingdom level, WGS identified 312 archaeas, 6,843 bacteria, 660 viruses, and 127 eukaryotes. The abundance information across all samples for each species was calculated and ranked to identify the most abundant species, which is the default parameter implemented in Kraken2, using MEGAN6 to obtain the taxonomic profile in kingdom, phylum and genus. We utilized MEGAN6 to visualize the abundance information of all identified microorganisms at the phylum level in a radial tree chart. This visualization provided a comprehensive view of the microbial composition across all 172 pediatric infection samples, highlighting the relative abundance of different phyla (Supplementary Figure S2). We generated a Taxonomy Rarefaction Plot to assess the microbial diversity in our study. This plot provides valuable insights into the richness and evenness of the microbial taxa across the sampled pediatric infection dataset. As shown in the plot, the rarefaction curve reaches a plateau, indicating that the sampling effort has adequately captured the majority of the microbial diversity present in the samples. This analysis highlights the comprehensive nature of our dataset and strengthens the reliability of our findings (Supplementary Figure S3). After filtering the host sequences of the raw data with quality control and criteria (Method), we identified a total of 7,943 OTUs in this dataset, with an average of 46.18 (*n* = 172) OTUs per sample.

### 3.3 Batch effects in taxonomic profiles of pediatric infection samples from diverse data sources

**Figure 1:**
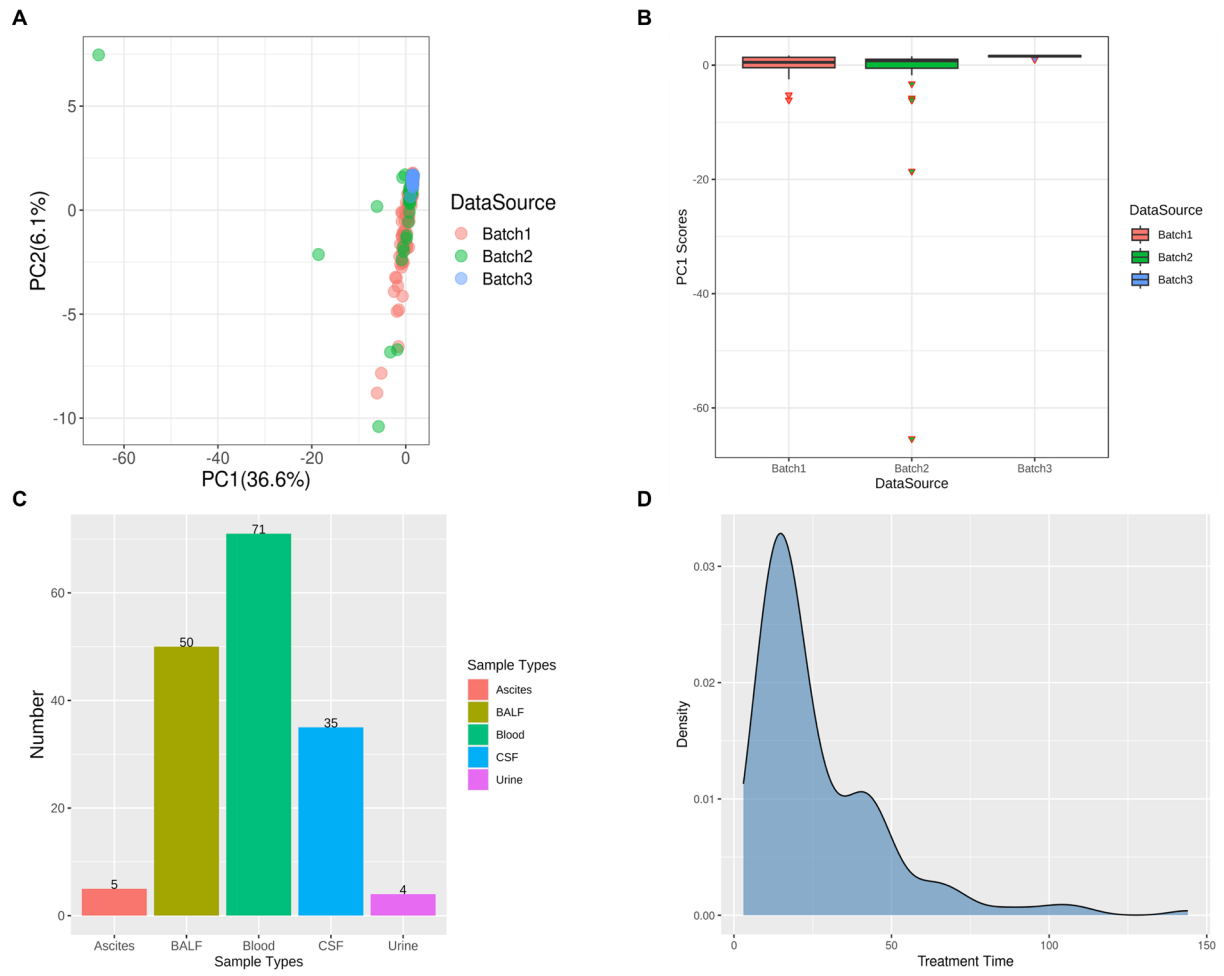
Principal Component Analysis (PCA) and Outlier Removal. **(A)** PCA Results Delineated by Data Source: Each batch is uniquely represented by a color scheme, with red, green, and blue corresponding to three distinct batches. **(B)** Identification of Outliers Based on Principal Component 1 (PC1) Scores: The outliers in each batch are represented by inverted triangle symbols, with the batches themselves distinguished by the same color scheme as in **(A)** – red, green, and blue. **(C)** Post-Outlier Removal Distribution of Samples by Sample Type: Different sample types are color-coded for clarity. Red signifies ascites, yellow-green denotes bronchoalveolar lavage fluid (BALF), green represents blood, blue is indicative of cerebrospinal fluid (CSF), and purple corresponds to urine. **(D)** Distribution of Treatment Time in All Samples.

Based on the taxonomic profiles from Karken2 and MEGAN6, the principal component analysis was performed on the abundance information of all identified microorganisms in all 172 samples in each phylum (Figure 1A). From the figure of PCA, it can be seen that some samples are far away from most of the samples, and these samples contribute a lot to the first component. In total, six samples contributed abnormally to the first principal component, which means that these samples may show the batch effects compared to the other samples, so these anomalous sample points should be removed and not included in the subsequent analysis (Figure 1B). Upon examining the clinical information of these sample outliers, it was observed that these samples were from cohorts 1, 2, and 3, with the sample types being bronchoalveolar lavage fluid (BALF), cerebrospinal fluid (CSF), and blood, respectively. Out of the six patients from whom these samples were sourced, five were under the age of four. All of these patients received antibiotics during their hospitalization and exhibited improved outcomes upon discharge (Supplementary Table S3). However, these six samples are planned to be excluded from further analysis. After excluding these outlier samples, the distribution of sample types and sources of infection in the remaining dataset can be observed in the (Figures 1C,D).

### 3.4 The existence of *Cryptococcus*, *Citrobacter* and *Colwellia* significantly associates with high costs and prolonged treatment duration in pediatric infections

**Figure 2:**
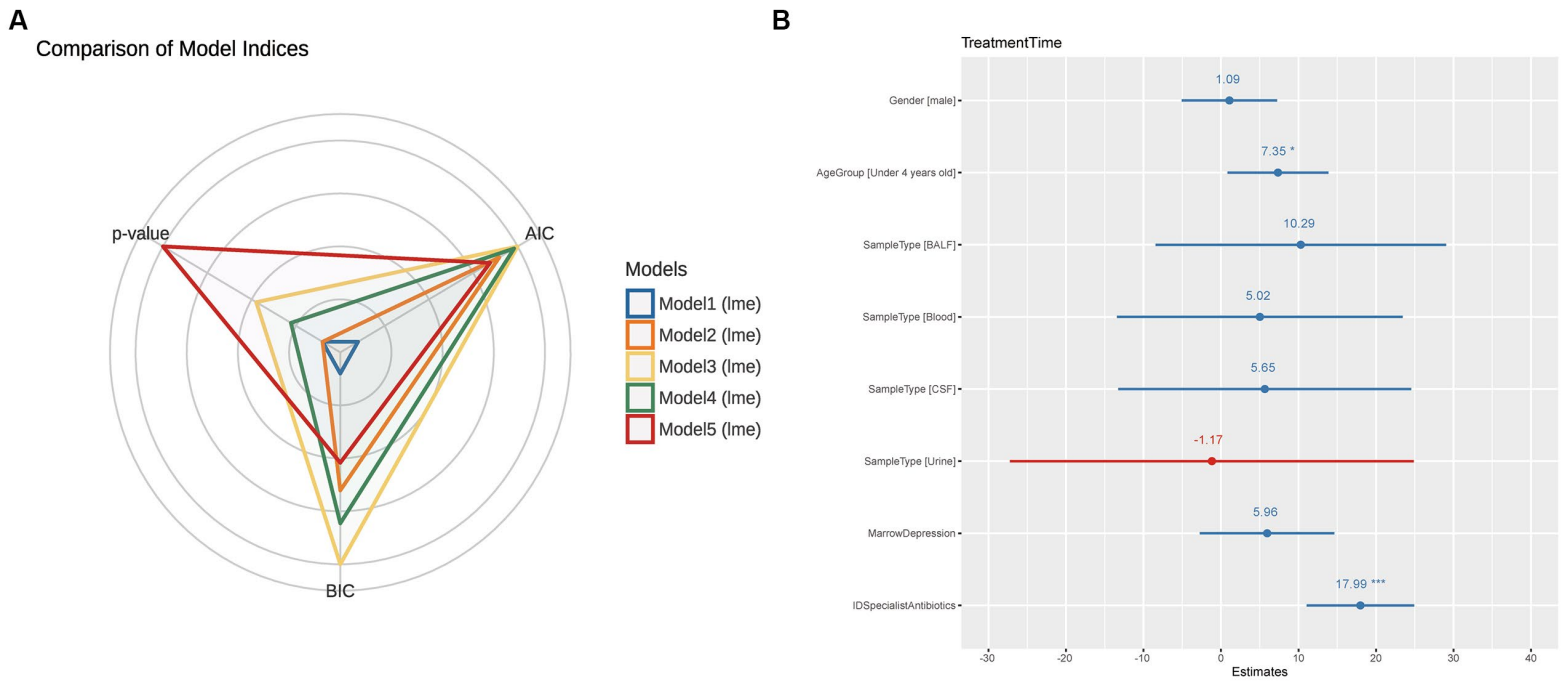
Comparison of Statistical Models and Summary of the Optimal Model. **(A)** This figure compares five different statistical models using radar charts, where each axis represents a model and the distance from the center corresponds to the Akaike information criterion (AIC) and Bayesian information criterion (BIC) values. Each model is color-coded as follows: Blue (Model 1): TreatmentTime ~ Gender + AgeGroup + SampleType + MarrowDepression. Orange (Model 2): TreatmentTime ~ Gender + AgeGroup + SampleType + MarrowDepression + IDSpecialistAntibiotics. Yellow (Model 3): TreatmentTime ~ Gender + AgeGroup + MarrowDepression + IDSpecialistAntibiotics + AntibioticTreatment. Green (Model 4): TreatmentTime ~ Gender + AgeGroup + MarrowDepression + IDSpecialistAntibiotics + AntibioticTreatment + MetagenomicResults. Red (Model 5): TreatmentTime ~ Gender + AgeGroup + MarrowDepression + IDSpecialistAntibiotics + AntibioticTreatment + MetagenomicResults + CultureResults. The proximity to the center indicates the model’s performance, with a closer distance representing better performance. Among the models, the orange model (Model 2) performed the best, exhibiting the lowest AIC and BIC values and significant *p*-values. **(B)** After selecting Model 2 as the best model, this figure provides a comprehensive summary of the model. As described in the Methods section, Model 2 includes age group, gender, sample type, marrow depression, and ID specialist antibiotic use as fixed effects, with data source considered as a random effect. The summary offers detailed information on coefficients, standard errors, *z*-values, and *p*-values associated with each factor, providing a complete overview of the model’s performance and statistical significance.

**Figure 3:**
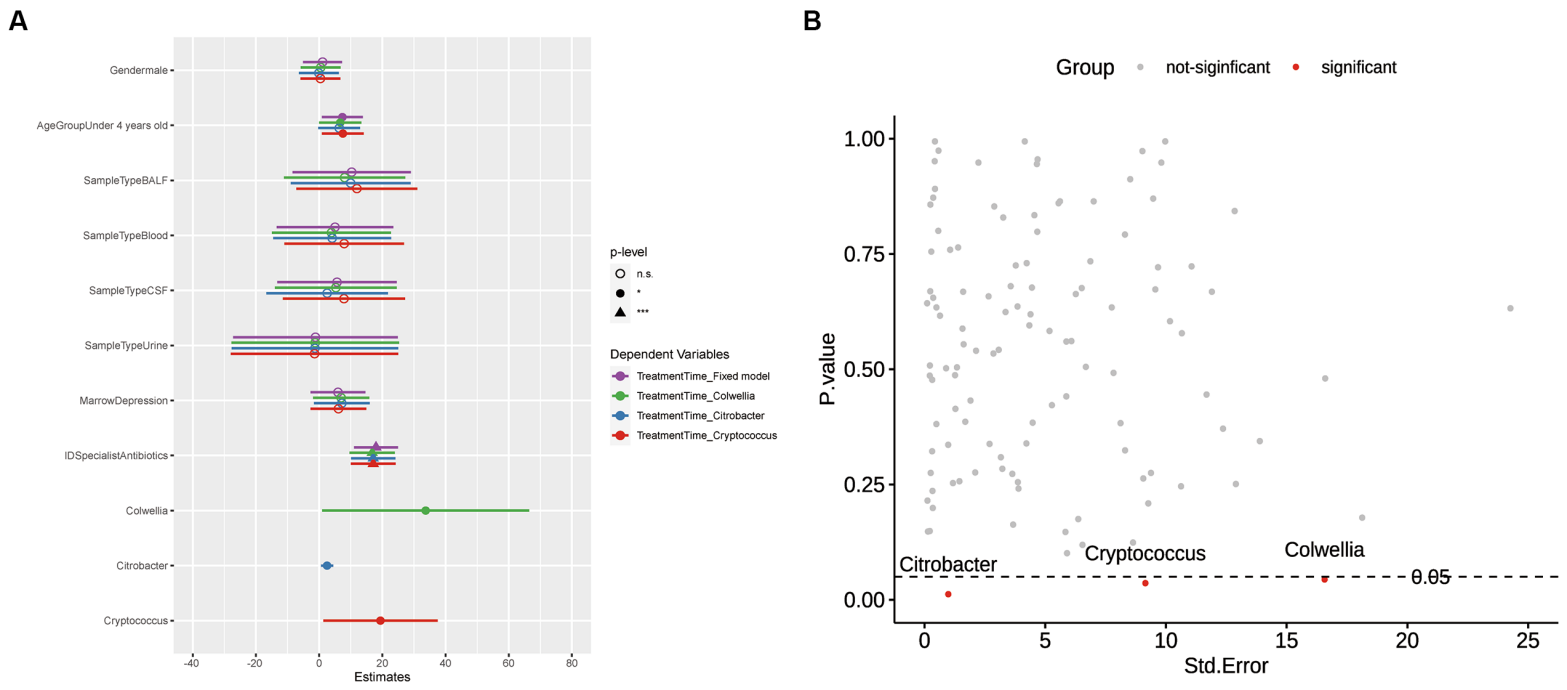
Visualization of *p*-values and Model Comparisons. **(A)** Scatterplot of *p*-values versus Standard Errors: This plot visualizes the *p*-values corresponding to each Genus in the model. The color scheme is used to indicate significance, with red denoting significance and gray indicating non-significance. **(B)** Comparison of Models Derived from Significant Microorganisms: The models are color-coded to represent different scenarios. Purple represents a fixed model without the inclusion of microorganisms. Green, blue, and red, respectively, represent models that include Colwellia, Citrobacter, and Cryptococcus. Regarding the *p*-values, the significance levels are annotated as follows: Asterisks denote statistical significance levels (*p < 0.05, **p < 0.01, ***p < 0.001). Additionally, non-significant results (*p* ≥ 0.05) are denoted as “n.s.” (not statistically significant).

In Figure 2A, we compared multiple models and selected Model 2, which demonstrated the lowest AIC and BIC values and a significant value of *p*. The final best-fit model, as mentioned in the methods section, incorporated age group, gender, sample type, and the use of ID specialist antibiotics as fixed effects, while the batch was included as a random effect. The estimates of this optimal model is presented in Supplementary Table S4 and Figure 2B. Utilizing the final best-fit model, we incorporated each genus into the analysis. This allowed us to screen for microorganisms that were both relevant and clinically significant for our variables of interest (Supplementary Figure S4). Our results revealed that *Colwellia*, *Cryptococcus*, and *Citrobacter* all had a statistically significant impact on treatment time according to the linear mixed models (Figure 3A). Moreover, we compared the new models constructed by incorporating each of these three microorganisms. As depicted in Figure 3B, all three microorganisms exert a significant influence on the treatment time, which is our primary concern.

The genus *Colwellia*, which includes some of the most extreme cold-loving and pressure-tolerant species known to date, are heterotrophic and facultatively anaerobic. They thrive in persistently cold marine environments, such as the sea ice of the Arctic and Antarctic. *Colwellia* species have been isolated from organic-rich marine habitats, including sediments or homogenates originating from marine invertebrates like amphipods and squid, or vertebrates like fish (Peoples et al., 2020). However, the microorganisms of *Cryptococcus* and *Citrobacter* spp., are more associated with infections. *Cryptococcus* mainly causes meningoencephalitis, as well as other diseases affecting the central nervous system (CNS) and lungs, and is associated with severe morbidity (Chen et al., 2014; Setianingrum et al., 2019). *Citrobacter* spp. belong to a group of parthenogenic, anaerobic, gram-negative rods in the Enterobacteriaceae family. They are frequently found in water, soil, food, and the intestinal tract of animals and humans. Previously considered low virulence environmental contaminants or colonizing bacteria, they are now known to cause a wide range of infections, including urinary tract, liver, biliary tract, peritoneum, intestine, bone, respiratory tract, endocardium, wounds, soft tissues, meninges, and blood (Liu et al., 2018; Lee et al., 2019; Liu et al., 2021).

### 3.5 Significant association between abundance of *Colwellia*, *Cryptococcus*, and *Citrobacter*, and multiple clinical indicators in severe infections

**Figure 4:**
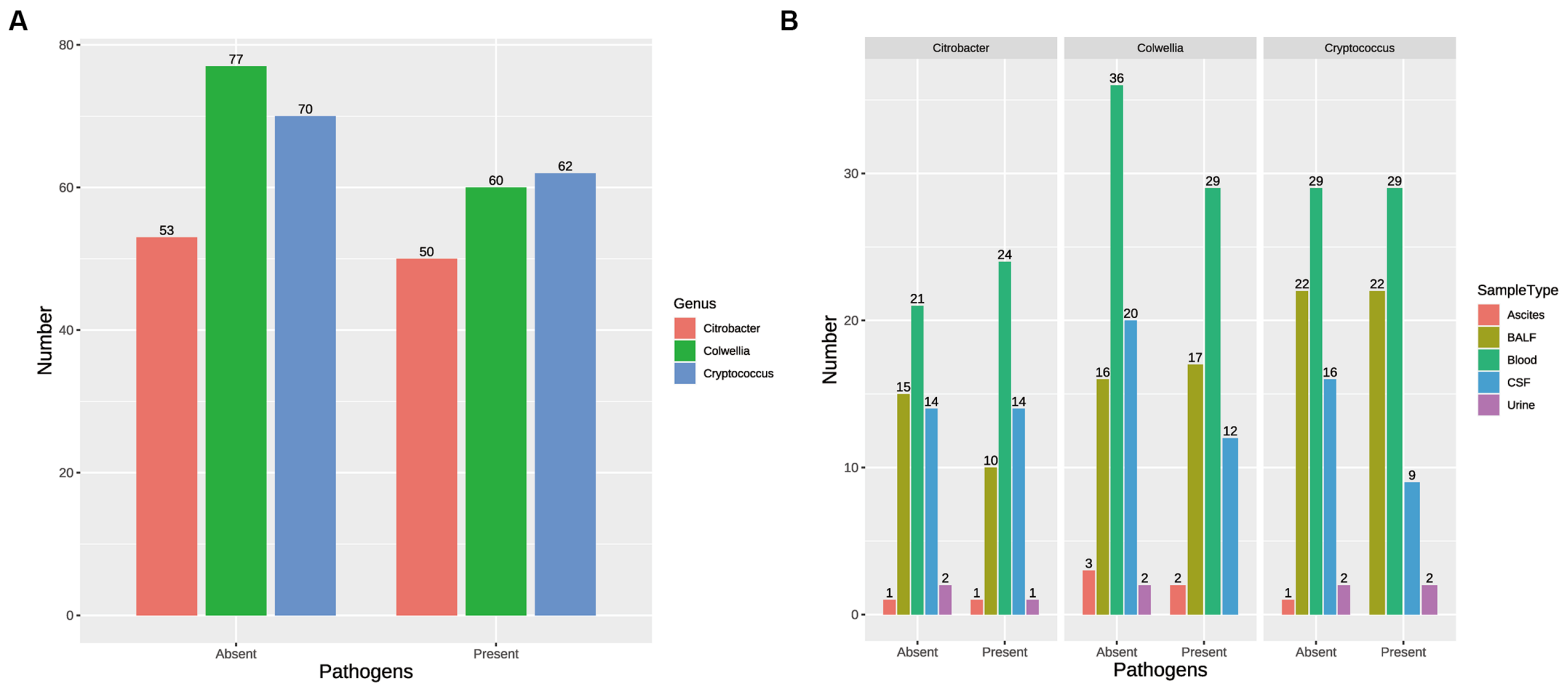
Impact of Pathogen Presence on Sample Distribution. **(A)** Number of Samples Post-Propensity Score Matching (PSM): This part of Supplementary Figure S7 presents the number of samples, after propensity score matching, that are either infected with or free from the three pathogens (Colwellia, Cryptococcus, and Citrobacter). The samples are categorized based on the presence or absence of these pathogens. **(B)** Distribution of Samples across Different Sample Types: This part of Supplementary Figure S7 displays the distribution of samples across different sample types, such as blood, bronchoalveolar lavage fluid (BALF), cerebrospinal fluid (CSF), urine, and ascites. The samples in each sample type are further categorized based on the presence or absence of the three pathogens (Colwellia, Cryptococcus, and Citrobacter).

Propensity score matching (PSM) was employed to evaluate the causal impact of three previously identified microorganisms (*Colwellia*, *Cryptococcus*, *Citrobacter*) on outcomes. The presence or absence of these pathogens was transformed into binary variables to indicate their presence or absence. Gender, age group, and sample type were used as covariates to balance the data between the treatment and control groups. Following matching, the Standardized Mean Difference (SMD) in the distribution of covariates between the two groups mostly remained below the threshold of 0.1, indicating high matching quality (Supplementary Figures S5A–C). The similarity in the distribution of covariates between the treatment and control groups is visually demonstrated through jitter plots (Supplementary Figures S5D–F) and histograms of propensity score distribution (Supplementary Figures S5G–I). The matching process helps to mitigate potential confounding factors, ensuring a more accurate assessment of the causal impact of *Cryptococcus*, *Citrobacter* and *Colwellia* on the treatment outcomes and clinical metrics of pediatric infections. Figure 4 is composed of two parts. Figure 4A presents the number of samples, post-propensity score matching (PSM), that are either infected with or free from the three pathogens. Additionally, Figure 4B displays the distribution of samples across different sample types, each categorized based on the presence or absence of the three pathogens.

Subsequently, the newly constructed dataset was analyzed to investigate the impact of these pathogens’ presence or absence on treatment time and other clinical metrics using the matched dataset. We performed a reanalysis of the matched dataset obtained through propensity score matching (PSM) and explored the associations between the presence or absence of these three pathogens and various clinical indicators using Wilcoxon rank-sum tests.

The reanalyzed results revealed significant differences in the duration of treatment, total cost of hospitalization, and overall hospitalization expenses between the groups with and without these microorganisms (Supplementary Table S5). Additionally, the presence or absence of these microorganisms was found to be associated with lymphocyte percentage (LY%), neutrophil percentage (NE%), hemoglobin levels (HGB), platelet count (PLT), and white blood cell count (WBC).

**Figure 5:**
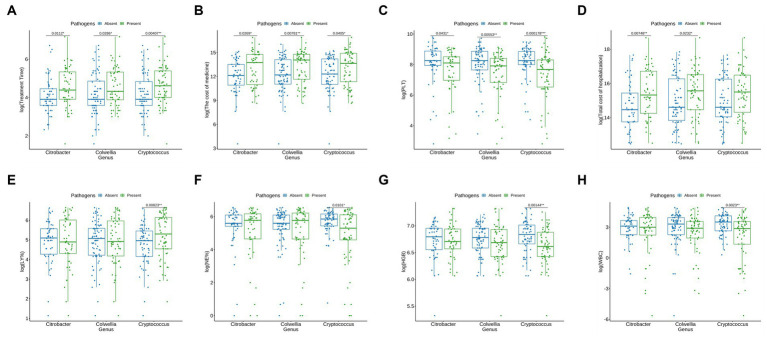
Influence of Microorganisms on Clinical Parameters and Costs. **(A)** Influence on Treatment time (days): A significant extension in treatment duration is observed in patients with Cryptococcus, Citrobacter, and Colwellia (represented by green bars) in comparison to those without these microorganisms (blue bars). **(B)** Impact on Medication Costs (RMB): The presence of Cryptococcus, Citrobacter, and Colwellia is associated with a substantial elevation in medication costs (green bars) when compared to patients not harboring these microorganisms (blue bars). **(C)** Effect on Platelet Count (PLT) (x 10^9/L): Patients with Cryptococcus, Citrobacter, and Colwellia exhibit a noticeable reduction in platelet count (PLT) (green bars) relative to those without these microorganisms (blue bars). **(D)** Influence on Hospitalization Expenses (RMB): The hospitalization costs are significantly higher for patients with Citrobacter and Colwellia (green bars) compared to those without these microorganisms (blue bars). **(E)** Impact on Lymphocyte Percentage (%): A significant increase in lymphocyte percentage (LY%) is noted in patients with Cryptococcus (green bars) as opposed to patients without this pathogen (blue bars). **(F)** Effect on Neutrophil Percentage (%): Patients with Cryptococcus manifest a significant reduction in neutrophil percentage (NE%) (green bars) in comparison to patients without this pathogen (blue bars). **(G)** Influence on Hemoglobin (HGB) Levels (g/L): A significant decrease in hemoglobin (HGB) levels is observed in patients with Cryptococcus (green bars) relative to patients without this pathogen (blue bars). **(H)** Impact on White Blood Cell Count (WBC) (x 10^9/L): Patients with Cryptococcus demonstrate a significant reduction in white blood cell count (WBC) (green bars) compared to patients without this pathogen (blue bars). These findings suggest that the presence of Cryptococcus, Citrobacter, and Colwellia is linked with adverse clinical outcomes, such as extended treatment duration, elevated medication costs, reduced platelet count, and increased hospitalization expenses. The significant alterations in lymphocyte percentage (LY%), neutrophil percentage (NE%), hemoglobin (HGB), and white blood cell count (WBC) further indicate potential impacts on the overall health status of patients harboring these microorganisms.

In our findings, the presence of *Cryptococcus*, *Citrobacter*, and *Colwellia* significantly extended the treatment duration for patients (Figure 5A), resulted in a substantial increase in medication costs (Figure 5B), and caused a noticeable decrease in platelet count (PLT) (Figure 5C). Patients with *Citrobacter* and *Colwellia* also had significantly higher hospitalization costs (Figure 5D). Furthermore, compared to patients without these pathogens, those with *Cryptococcus* showed a significant increase in lymphocyte percentage (LY%), while their neutrophil percentage (NE%), hemoglobin (HGB), and white blood cell count (WBC) significantly decreased (Figures 5E–H).

These findings suggest that the co-occurrence of *Cryptococcus*, *Citrobacter* and *Colwellia* with the pathogenic organism responsible for the severe infection may contribute to prolonged hospital stays and increased treatment costs. Especially, the presence of *Cryptococcus* is significantly correlated with multiple clinical indicators, which may signify a potential impact on the overall health status of patients. This highlights the complexity and multifaceted nature of pediatric infections, emphasizing the importance of considering the presence of these microorganisms in the context of co-infections to better understand and manage the disease course effectively.

## 4 Discussion

While this study provides valuable insights, it is important to acknowledge its limitations. Firstly, the small sample size and single-center design may limit the generalizability of the findings. Conducting future studies with larger cohorts and multiple centers would be beneficial to validate and extend these results. Additionally, the retrospective nature of the study and the relatively short duration of data collection may introduce biases and restrict the depth of analysis. The identified pathogens in our study are not primary causative agents of the patients’ infections; all three of these pathogens are considered background pathogens. Speculations regarding their association with prolonged hospitalization and the use of ID specialist antibiotics include: (a) These three pathogens may potentially participate in influencing the formation of biofilms, thereby affecting the host’s defense mechanisms or influencing the drug resistance of colonized bacteria (Van Baarlen et al., 2007; Domínguez-Díaz et al., 2019; Mirzaei et al., 2020). (b) The production of endotoxins or peptides by these three pathogens might lead to the depletion or reduction of cellular ATP levels, thereby enhancing the pathogen’s tolerance to antibiotics (Huemer et al., 2020). (c) These three pathogens may release corresponding endotoxins during the host’s diseased state, influencing the release of inflammatory factors and sustaining inflammation (Domínguez-Díaz et al., 2019; Rippon et al., 2022). (d) These three pathogens could potentially impact the host’s metabolomics, such as carbon or nitrogen sources, thereby enhancing the virulence of pathogenic bacteria (Rohmer et al., 2011). In the future, prospective studies could be conducted to investigate the relevant biological mechanisms associated with severe pediatric infections. By incorporating control and experimental groups, these studies would provide a more comprehensive understanding of the relationships between microorganism co-infections and clinical outcomes. By exploring the biological functions and interactions of these microorganisms, researchers can gain insights into the pathogenesis of severe infections in children. This knowledge can contribute to the development of targeted interventions and improved patient management strategies.

The findings presented here open up several avenues for future research. Firstly, it is crucial to understand the mechanisms underlying the observed associations between microorganism co-infections and clinical outcomes. Investigating the interplay between these microorganisms and the host immune response could shed light on the pathogenesis of severe pediatric infections. Secondly, exploring the impact of different treatment strategies on outcomes in the context of co-infections could guide the development of tailored therapeutic approaches. Moreover, further investigation is warranted to determine the potential role of these microorganisms as prognostic markers or targets for interventions, aiming to improve patient management and outcomes. The results of this study hold significant clinical implications. Identification of *Colwellia*, *Cryptococcus*, and *Citrobacter* in co-infections with pathogenic organisms appears to be associated with prolonged hospital stays and altered clinical indicators in severe pediatric infections. Recognizing the potential impact of these microorganisms could aid healthcare professionals in early risk assessment and appropriate treatment planning for affected patients. By considering the presence of these microorganisms in the clinical decision-making process, medical resources can be better allocated to improve patient care and optimize healthcare resource utilization.

While this study focused on a specific cohort of pediatric patients with severe infections, the results may have broader relevance. However, caution should be exercised when extrapolating the findings to other populations or infection types. Future studies encompassing diverse patient groups and a wide range of infectious diseases will be necessary to confirm the general applicability of these conclusions.

## 5 Conclusion

Although *Cryptococcus*, *Citrobacter*, and *Colwellia* are recognized as pathogens, they were not identified as the primary causative agents in the analyzed cases. However, the results of this study suggest that the co-infection of these three microorganisms with disease-causing organisms may significantly impact the treatment course in patients with severe pediatric infections. Especially, the presence of *Cryptococcus* was found to be significantly associated with prolonged treatment duration and multiple clinical indicators. These findings highlight the potential role of *Cryptococcus* in particular, in contributing to longer hospital stays, and altered clinical indicators. It suggests that the presence of these microorganisms in co-infections may contribute to the complexity and severity of pediatric infections, warranting additional medical attention and allocation of resources.

## Data availability statement

The original contributions presented in the study are publicly available. This data can be found at: https://www.ncbi.nlm.nih.gov/; PRJEB67633.

## Ethics statement

Ethical approval was not required for the study involving human samples in accordance with the local legislation and institutional requirements because [reason ethics approval was not required]. Written informed consent for participation in this study was provided by the participants’ legal guardians/next of kin. Written informed consent was obtained from the individual(s) for the publication of any potentially identifiable images or data included in this article.

## Author contributions

CL: Formal analysis, Methodology, Software, Validation, Visualization, Writing – original draft. JS: Data curation, Formal analysis, Methodology, Writing – original draft. QC: Data curation, Methodology, Writing – review & editing. DG: Data curation, Investigation, Methodology, Writing – review & editing. QL: Conceptualization, Investigation, Methodology, Project administration, Software, Supervision, Writing – review & editing. YY: Conceptualization, Investigation, Project administration, Supervision, Writing – review & editing.
