# Supplementary figures and images for: Whole-genome shotgun sequencing unravels the influence of environmental microbial co-infections on the treatment efficacy for severe pediatric infectious diseases
Source: Front Microbiol. 2024 Jan 24;15:1308871. doi: 10.3389/fmicb.2024.1308871 (PMC10847551; doi:10.3389/fmicb.2024.1308871)

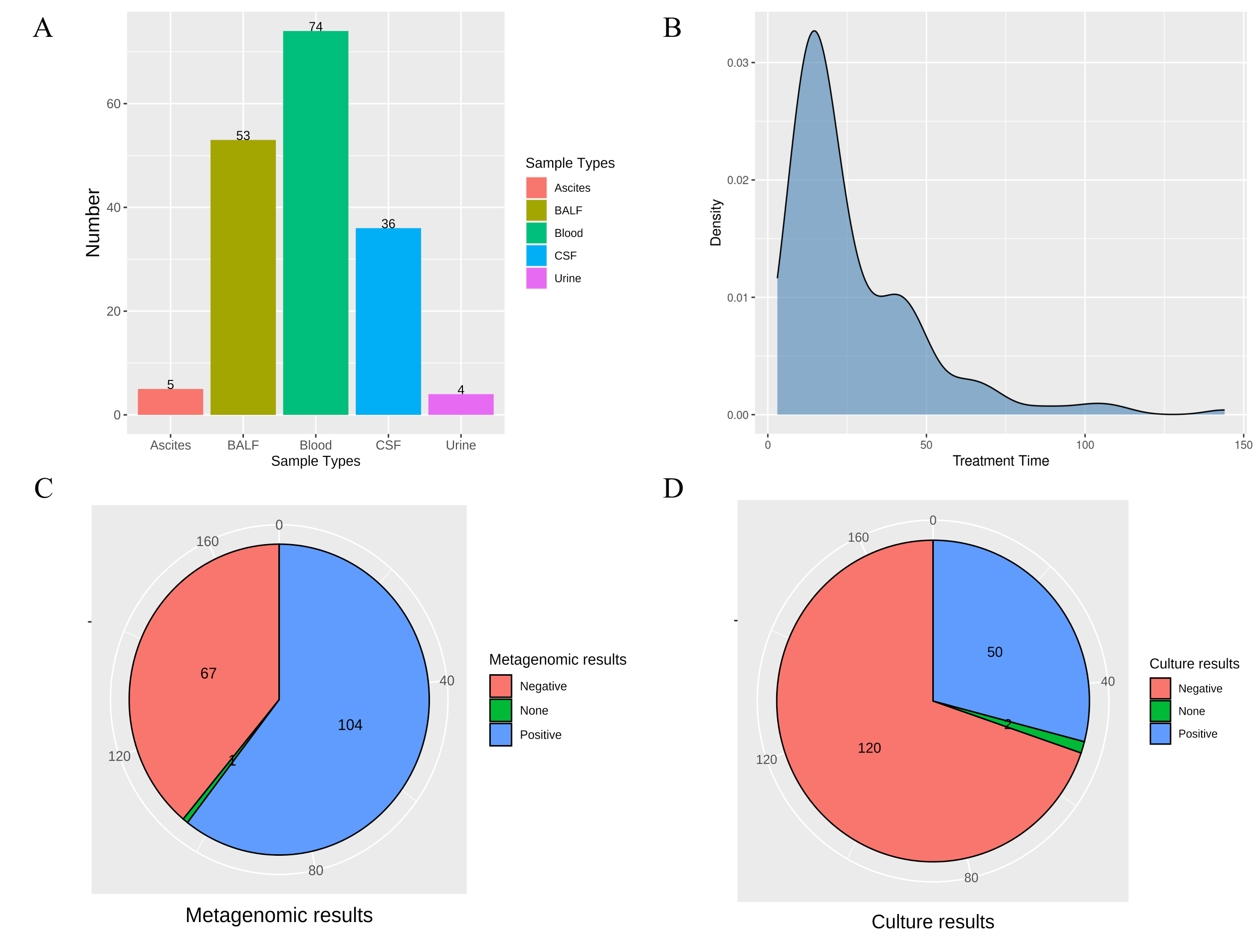

Supplement: Supplementary file 2 [file Image_1.TIFF]

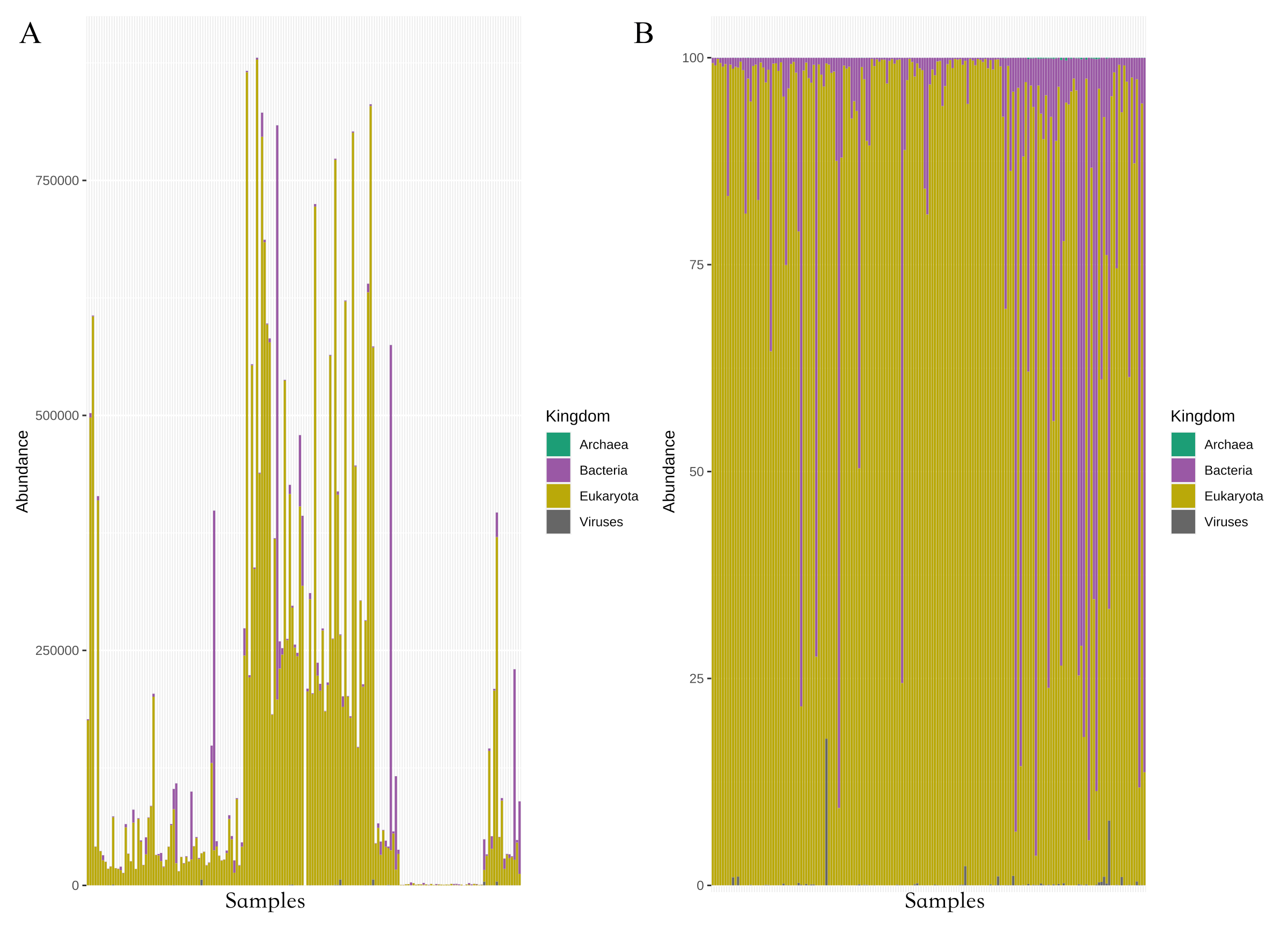

Supplement: Supplementary file 3 [file Image_2.TIFF]

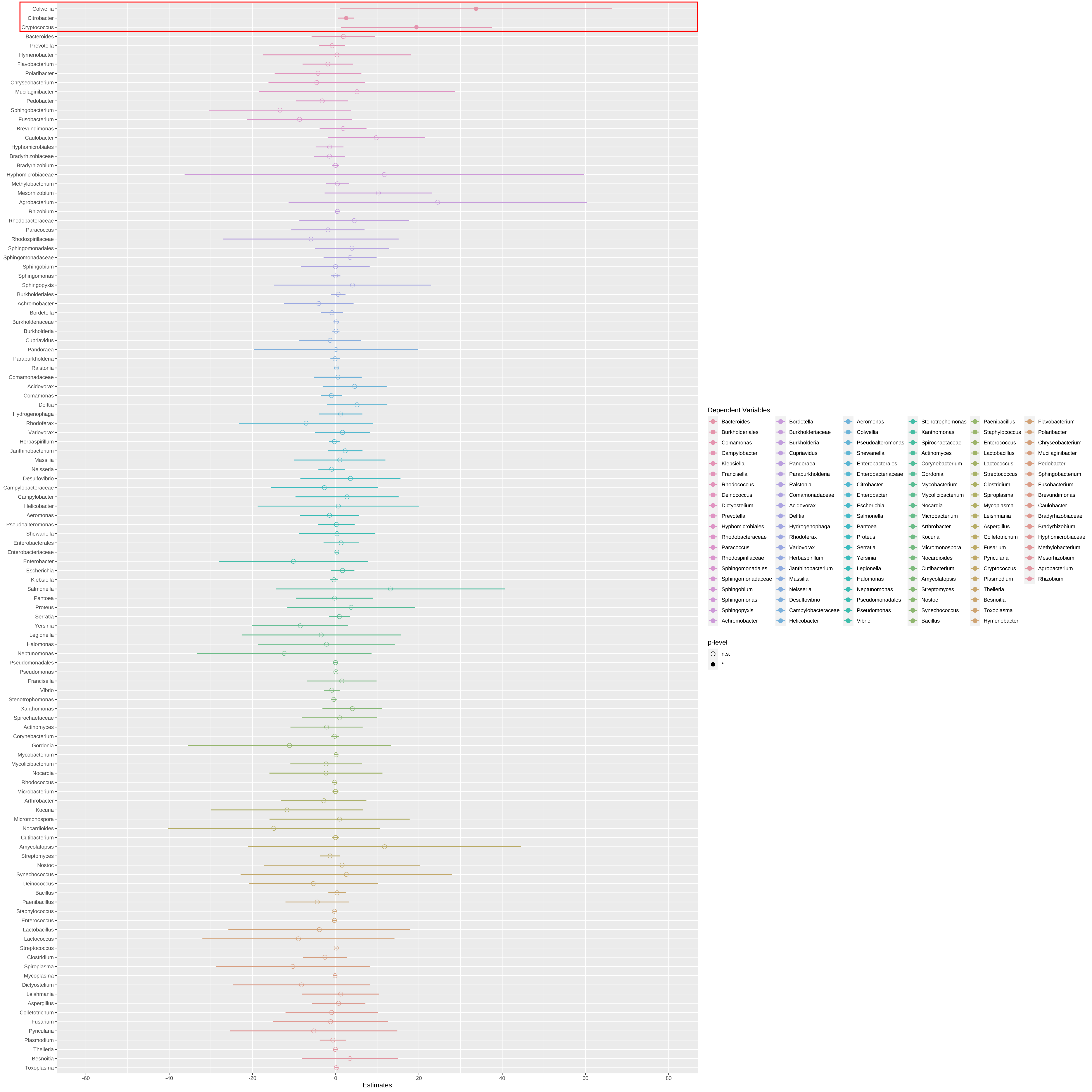

Supplement: Supplementary file 5 [file Image_4.TIFF]

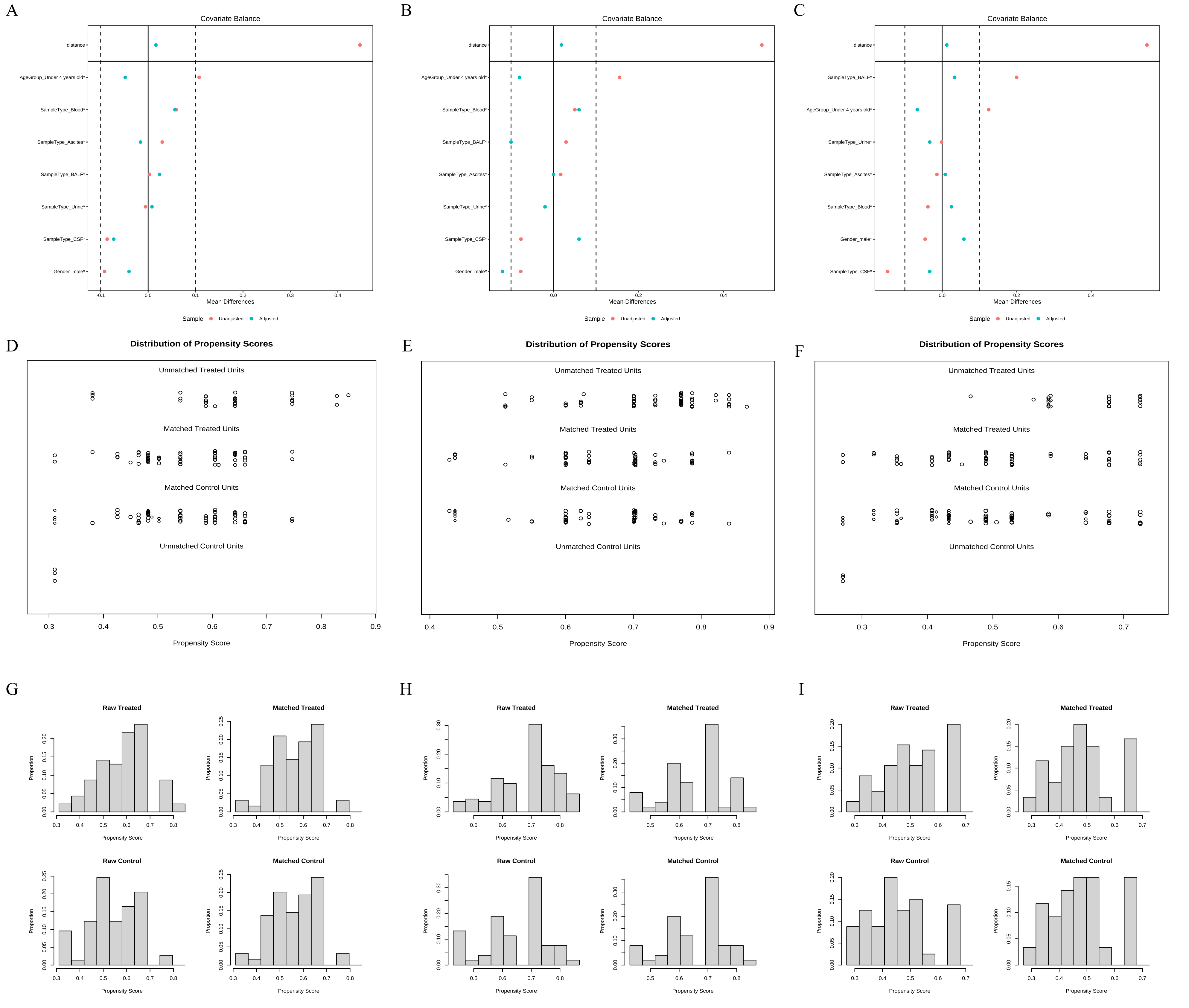

Supplement: Supplementary file 6 [file Image_5.TIFF]
